# Determination of Plasma Heparin Level Improves Identification of Systemic Mast Cell Activation Disease

**DOI:** 10.1371/journal.pone.0124912

**Published:** 2015-04-24

**Authors:** Milda Vysniauskaite, Hans-Jörg Hertfelder, Johannes Oldenburg, Peter Dreßen, Stefan Brettner, Jürgen Homann, Gerhard J. Molderings

**Affiliations:** 1 Institute of Exp. Haematology & Transfusion Medicine, University Hospital of Bonn, Bonn, Germany; 2 Department of Internal Medicine, St. Franziskus Hospital, Eitorf, Germany; 3 Department of Oncology, Hematology and Palliative Care, County Hospital, Waldbröl, Germany; 4 Community Hospital St. Elisabeth, Bonn, Germany; 5 Institute of Human Genetics, University Hospital of Bonn, Bonn, Germany; University of Kansas Medical Center, UNITED STATES

## Abstract

Diagnosis of *mast cell activation disease* (MCAD), i.e. *systemic mastocytosis* (SM) and *idiopathic systemic mast cell activation syndrome* (MCAS), usually requires demonstration of increased mast cell (MC) mediator release. Since only a few MC mediators are currently established as biomarkers of MCAD, the sensitivity of plasma heparin level (pHL) as an indicator of increased MC activation was compared with that of serum tryptase, chromogranin A and urinary N-methylhistamine levels in 257 MCAD patients. Basal pHL had a sensitivity of 41% in MCAS patients and 27% in SM patients. Non-pharmacologic stimulation of MC degranulation by obstruction of venous flow for 10 minutes increased the sensitivity of pHL in MCAS patients to 59% and in SM patients to 47%. In MCAS patients tryptase, chromogranin A, and N-methylhistamine levels exhibited low sensitivities (10%, 12%, and 22%, respectively), whereas sensitivities for SM were higher (73%, 63%, and 43%, respectively). Taken together, these data suggest pHL appears more sensitive than the other mediators for detecting systemic MC activity in patients with MCAS. The simple, brief venous occlusion test appears to be a useful indicator of the presence of pathologically irritable MCs, at least in the obstructed compartment of the body.

## Introduction

*Systemic mast cell activation disease* (MCAD) denotes a group of primary mast cell (MC) disorders characterized by aberrant release of variable subsets of MC mediators due to certain sets of genetic mutations sometimes also leading to accumulation of dysfunctional MCs in potentially any organs and tissues [1, 2]. According to current proposed classifications of MCAD [1,3,4], the traditionally recognized rare variant termed *systemic mastocytosis* (SM) is characterized by specific constitutively activating somatic mutations in exon 17 of the tyrosine kinase KIT and immunohistochemical findings (known as the *World Health Organization (WHO) criteria*) [5] caused by these mutations. SM is further divided into several subtypes [6]. The other variant, only recently recognized, is termed *idiopathic MC activation syndrome* (MCAS). Like SM, MCAS is seemingly born of sets of mutations in various genes (for review, see [2]) and presents a complex clinical picture of multiple MC mediator-induced symptoms, but unlike SM, the mutations in MCAS seem to drive relatively little MC proliferation and MCAS patients fail to meet the WHO criteria for diagnosis of SM [1,3,4]. While the prevalence for SM has been calculated to vary between 0.3:100,000 (Germany) [7], 9.59:100,000 (Denmark) [8] and 13:100,000 (Netherlands) [9], the prevalence for MCAS may be as high as 5–10% (Germany). [10] Hence, MCAS is a common disease.

Diagnosis of MCAD usually involves demonstrating increased MC activation, e.g., MC mediator release [1,4,11]. Currently, however, only a few (Table 1) of the more than 200 mediators synthesizable by MCs are assessable in the clinical laboratory to detect MC activation: tryptase, histamine, and chromogranin A (CgA) in serum, and leukotrienes, prostaglandin D_2_ (PGD_2_) and/or its metabolite 9α,11β-PGF_2α_, and N-methylhistamine (NMH) in urine (Table 1). We have provided preliminary evidence that plasma heparin level (pHL) might also be a useful biomarker for MC activation [12]. The aim of the present study was to determine in a large cohort of MCAD patients the sensitivity of pHL as an indicator of increased MC activation and to compare its sensitivity with those determined in the same patients for tryptase and CgA in serum (sTryp and sCgA, respectively) and NMH in urine (uNMH). We show that pHL indeed is more sensitive for systemic MC activation in patients with MCAS, but not in patients with SM, than sTryp, sCgA, and uNMH.

**Table 1:** Mast cell mediators or their metabolites in blood or urine which currently can be determined as routine laboratory parameters.

Increased level of the mediator or its metabolites	Sensitivity	Specificity for mast cells
***Tryptase*** level in blood > 20 ng/ml	**SM:** ~ 80–85% ([16]; further references therein); 77% [39]	in the absence of hematologic malignancies and end-stage kidney disease specific for mast cells; 10% falsely elevated results due to interference with rheumatoid factor [45]
	**MCAS:** 8% [40]; 22% [41]; 0% [42, 43]; 21% [39]; 33% [44]	
***N-Methylhistamine*** level in 24-hour urine	**SM:** ~ 50% [30]; 71% [57]; 81% [16]; 76% [39];	histamine is produced and released by basophils in addition to mast cells; uptake of histamine from food
	**MCAS:** 0% [42, 46]; 95% [47]; 18% [39]; 10% [44]	
***Serotonin*** level in blood	**SM:** 0% to 34% [48, 49]	mainly stored in enterochromaffin cells, serotonergic neurons and platelets; small amounts are present also in mast cells (for review, see [50])
	**MCAS:** 0% [49]	
Levels of ***leukotrienes*** in urine	**SM:** ~ 50% [51]; 44% [52]	produced by many cell types
Level of ***prostaglandin D***_***2***_ and its metabolites in urine	**SM:** 94% [57]; 100% [53]; 62% [56];	predominantly produced by mast cells [53, 54]; small quantities are also formed by basophils, eosinophils, Th2-lymphocytes and macrophages [55]
	**MCAS:** 75% [42], 68% [35]	

## Methods

### Patients

Data from 257 Caucasian patients (for details, see Table 2) presenting consecutively between May 2005 and December 2013 with MCAD diagnosed per current criteria [1,3,4,6] were included in this study. Diagnostic criteria of SM and MCAS as well as the summarized diagnostic findings of the patients are listed in Tables 3 and 4. Patient age ranged from 18 to 86 years (mean: 48.5 years; male to female ratio: 1:3.3). For diagnostic purposes, presence of MC mediator-related symptoms was assessed by a validated questionnaire [10,13]. For differential diagnosis, other diseases presenting similar symptoms were ruled out by appropriate assessments including laboratory testing, imaging, and/or endoscopy. KIT^D816V^ mutation status for SM patients was determined by polymerase chain reaction-based methods at commercial laboratories. During diagnostic investigation, patients were not taking MC-activity-regulating drugs and did not take proton pump inhibitors which would have influenced CgA levels. All data in this study were collected during routine clinical evaluations of MCAD patients who provided informed consent for use of such data in research. Patient information was anonymized and de-identified prior to analysis. As such, the Ethics Committee of the medical faculty of the Rheinische Friedrich-Wilhelms-University of Bonn classified this study as exempt from requiring specific patient consent.

**Table 2:** Characteristics of the study population.

total (n = 257)		MCAS (n = 238)		SM (n = 19)	
male (n = 58)	female (n = 199)	male (n = 52)	female (n = 186)	male (n = 6)	female (n = 13)
*age [years]*: *mean ± SD*, *median*, *range*		*age [years]*: *mean ± SD*, *median*, *range*		*age [years]*: *mean ± SD*, *median*, *range*	
47 ± 16, 47, 19–86	50 ± 14, 50, 18–81	46 ± 16, 45, 19–86	50 ± 14, 50, 18–81	59 ± 10, 56, 46–71	49 ± 10, 46, 33–72

Total number of patients included (total) in the present investigation, number of patients with idiopathic systemic mast cell activation syndrome (MCAS) and with systemic mastocytosis (SM). SD—standard deviation.

**Table 3:** Percentage of the study population fulfilling the criteria proposed to define *mast cell activation syndrome* (for references, see text) when all other diagnoses that could better explain the full range and chronicity of the findings in the case have been excluded.

Criteria proposed to define *mast cell activation syndrome*			Percentage
***Major criteria***			
1.	Focal or disseminated increased number of mast cells in marrow and/or extracutaneous organ(s) (e.g., gastrointestinal tract biopsies; CD117-, tryptase- and CD25-stained)		37% (96/257)
2.	Constellation of clinical complaints attributable to pathologically increased mast cell activity (mast cell mediator release syndrome)		100% (257/257)
***Minor criteria***			
1.	Abnormal spindle-shaped morphology in >25% of mast cells in marrow or other extracutaneous organ(s)		2% (2/257)
2.	Abnormal mast cell expression of CD2 and/or CD25 (i.e., co-expression of CD117/CD25 or CD117/CD2)		4% (11/257)
3.	Detection of genetic changes in mast cells from blood, bone marrow or extracutaneous organs for which an impact on the state of activity of affected mast cells in terms of an increased activity has been proven.		9% (23/257)
4.	Evidence (typically from body fluids such as whole blood, serum, plasma, or urine) of above-normal levels of mast cell mediators including:		
	•	tryptase in blood	13% (33/257)
	•	histamine or its metabolites (e.g., *N*-methylhistamine) in urine	21% (53/257)
	•	heparin in blood*	56% (143/257)
	•	chromogranin A in blood (potential confounders of cardiac or renal failure, neuroendocrine tumors, or recent proton pump inhibitor use were excluded)	11% (27/257)
	•	other relatively mast cell-specific mediators (e.g., eicosanoids including prostaglandin PGD_2_, its metabolite 11-β-PGF_2α_, or leukotriene E4)	21% (54/257)
5.	Symptomatic response to inhibitors of mast cell activation or mast cell mediator production or action (e.g., histamine H1 and/or H2 receptor antagonists, cromolyn)		56% (145/257)

The diagnosis *mast cell activation syndrome* is made upon fulfilment of either both major criteria or the second criterion plus at least one minor criterion. In parentheses the number of patients fulfilling the respective criterion over the total number of patients included in the present study is given. Differences between the percentages given for tryptase, heparin, chromogranin A and N-methylhistamine in the present table and the corresponding values in Table 5 for MCAS patients are due to the fact that in the present table findings from the medical histories have been considered in addition to those obtained in the present investigation.

* Heparin level in blood was not used in the present study to define the diagnose MCAS.

**Table 4:** Percentage of the study population fulfilling the criteria proposed to define *systemic mastocytosis* (for references, see text) when all other diagnoses that could better explain the full range and chronicity of the findings in the case have been excluded.

Criteria to define *systemic mastocytosis*	Percentage
**Major criterion**	
Focal dense infiltrates of mast cells (i.e., aggregates of >15 mast cells) in marrow and/or other extracutaneous organ(s) and confirmed by tryptase immunohistochemistry or other special stains	95%
	(18/19)
**Minor criteria**	
Abnormal or spindle-shaped morphology in >25% of mast cells in marrow or other extracutaneous organ(s)	42%
	(8/19)
Abnormal marrow mast cell expression of CD2 and/or CD25 (i.e., co-expression of CD117/CD25 or CD117/CD2)	47%
	(9/19)
KIT mutation at codon 816 in marrow, blood or extracutaneous organ(s)	79%
	(15/19)
Serum total tryptase > 20 ng/ml (does not apply in patients who have associated hematologic non-mast-cell lineage disease)	53%
	(10/19)

According to the WHO criteria, the diagnosis *systemic mastocytosis* is established if the major criterion and at least one minor criterion or at least three minor criteria are fulfilled. In parentheses the number of patients fulfilling the respective criterion over the total number of patients included in the present study is given.

Twenty-four healthy volunteers (inclusion criteria: absence of chronic or recurrent abdominal discomfort or disease during the last two years, non-smoker, no clinical signs for a malignant disease or mast cell mediator-related symptoms) who were gender-matched (median age 36 years, range 20–52 years, male to female ratio 1:3) were included in the study to investigate whether venous occlusion of the upper arm for 10 minutes (see below) induces an increase in the pHL in peripheral blood taken from a vein below the pressure cuff. Healthy subjects were recruited at the blood bank of the University Hospital of Bonn and gave informed consent as approved by the aforementioned Ethics Committee.

### Determination of heparin, tryptase, chromogranin A, and N-methylhistamine levels

Plasma heparin levels were determined by chromogenic anti-factor Xa (anti-Xa) assay (Siemens Healthcare, formerly Dade Behring) until January 2012 with an upper reference value of 0.02 anti-Xa IU/ml determined in-house. Thereafter, the COAMATIC Heparin assay (Chromogenix, Milano, Italy) was used with newly adapted reference ranges for determination of endogenous pHL with an upper reference value of 0.05 anti-Xa IU/ml also determined in-house. For heparin stabilization in plasma, CTAD tubes (S-Monovette 2.9ml CTAD, Sarstedt, Nümbrecht, Germany) were used. Because of the rapid degradation and neutralization of heparin at ambient temperature, blood specimens for pHL were kept continuously chilled at all stages of handling on ice or in a refrigerated environment. It is important that the blood specimen is centrifuged to obtain the plasma within 30 minutes of phlebotomy. In order to induce a standardized mild non-pharmacological stimulation of MCs in an easily accessible compartment of the body, we applied venous occlusion of the upper arm for 10 minutes, using a blood pressure cuff inflated 10 mm Hg above diastolic pressure. Hypoxia and increased compartment pressure induced by the stasis of blood flow in the arm would be expected to increase activation of pathologically irritable MCs in this compartment, leading to heparin release [12,14,15]. Venous blood samples were drawn (by standard brief tourniquet application) just before initiating venous occlusion (baseline) and just prior to ceasing venous occlusion. The venous occlusion test regularly provokes hemoconcentration of plasma proteins. Therefore, we compared the “heparin hemoconcentration ratio” (i.e., ratio of post-occlusion pHL to baseline pHL) to the fibrinogen hemoconcentration ratio.

sTryp and sCgA were determined from ice-cooled baseline blood samples. All sTryp assays were performed using the Unicap tryptase assay (Pharmacia Diagnostics, Uppsala, Sweden) with an upper reference level of 11.4 μg/l. uNMH was determined from 24-hour collections of urine (sent to the laboratory deep-frozen by the quickest mail) by liquid chromatography with mass spectrometry (LC-MS/MS). sCgA was detected by an immunoradiometric assay. sCgA and uNMH determinations were done at various commercial laboratories with different laboratory-specific reference values, so absolute values for these tests are not listed in S1 Table and instead we show only relative results (increased, normal, or decreased).

### Statistics

Correlation analysis of the data was performed by means of the program GraphPad Prism 4.

## Results

### Effect of venous occlusion on plasma heparin level

Similar to our previous findings [12], basal pHL from healthy people was 0.037 ± 0.006 anti-Xa IU/ml (n = 24; mean ± SEM), confirming the validity of our in-house upper reference level of 0.05 IU/ml. Venous occlusion did not induce a onsiderable increase in pHL. Likely due to hemoconcentration, post-occlusion pHL in the healthy subjects increased slightly to 0.043 ± 0.007 (n = 24; mean ± SEM).

### Sensitivity of levels of plasma heparin, serum tryptase and chromogranin A, and 24-hour urinary N-methylhistamine as biomarkers for MCAD

Basal pHL had a sensitivity for indicating increased MC activation of 41% in MCAS patients and 27% in SM patients (Table 5). Post-occlusion pHL (corrected for hemoconcentration) beyond the reference level was detected in 59% of the MCAS patients and in 47% of the SM patients.

**Table 5:** Sensitivities of heparin, tryptase, chromogranin A levels in blood and *N*-methylhistamine excretion into urine for indication of an increased mast cell activity.

**Heparin (basal value):**		
	all pts	98 pts/242 pts = 41%
	MCAS	94 pts/227 pts = 41%
	SM	4 pts/15 pts = 27%
**Heparin (basal plus after venous occlusion):**		
	all pts	141 pts/242 pts = 58%
	MCAS	134 pts/227pts = 59%
	SM	7 pts/15 pts = 47%
**Tryptase:**		
	all pts	27 pts/198 pts = 14%
	MCAS	18 pts/183 pts = 10% (> 20 ng/ml: 4 pts ≈ 2%)
	SM	11 pts/15 pts = 73% (> 20 ng/ml: 8 pts ≈ 53%)
**Chromogranin A (CgA):**		
	all pts	23 pts/155 pts = 15%
	MCAS	18 pts/147 pts = 12%
	SM	5 pts/8 pts = 63%
***N*-methylhistamine:**		
	all pts	33 pts/146 pts = 23%
	MCAS	30 pts/139 pts = 22%
	SM	3 pts/7 pts = 43%
**Heparin (basal plus after venous occlusion) plus tryptase:**		
	all pts	160 pts/257 pts = 62%
	MCAS	145 pts/238 pts = 61%
	SM	15 pts/19 pts = 79%
**Heparin (basal plus after venous occlusion) plus tryptase plus *N*-methylhistamine:**		
	all pts	169 pts/257 pts = 66%
	MCAS	154 pts/238 pts = 65%
	SM	15 pts/19 pts = 79%
**Heparin (basal plus after venous occlusion) plus tryptase plus *N*-methylhistamine plus CgA:**		
	all pts	174 pts/257pts = 68%
	MCAS	157 pts/238 pts = 66%
	SM	17 pts/19 pts = 90%

The sensitivity is given for each compound alone and for their combinations. pts—patients, MCAS—idiopathic systemic mast cell activation syndrome, SM—systemic mastocytosis. The differences between the number of patients listed in Table 2 and the numbers of patients in the subgroups given below is due to the fact that not all mediators were determined for each patient.

In MCAS patients sTryp exhibited low sensitivity (10%; Table 5). In fact, sTryp > 20 ng/ml (a minor diagnostic criterion for SM) was found in only 4 MCAS patients (2%). In contrast, elevated sTryp had a sensitivity of 73% in SM patients, and 53% of SM patients showed sTryp > 20 ng/ml (Table 5).

sCgA was elevated in 12% of the MCAS patients and 63% of the SM patients in this study (Table 5).

uNMH was elevated in 22% of the MCAS patients, whereas 43% of the SM patients excreted an increased amount of this histamine metabolite (Table 5).

pHL-based biomarker combinations were only slightly more sensitive than pHL alone for identifying MCAS (Table 5). In contrast, pHL-based biomarker combinations were nearly twice as sensitive (up to 90%) as pHL alone for identifying SM (Table 5).

### Correlation analysis of levels of plasma heparin, serum tryptase and chromogranin A, and 24-hour urinary N-methylhistamine

No correlations between any pairs of the MC mediators assessed in this study were apparent in the study population as a whole or the SM or MCAS subgroups.

## Discussion

In the present study the sensitivities of pHL, sTryp, sCgA, and uNMH as indicators of increased MC activation in MCAD patients were investigated. A key strength of our study is its inclusion of a large number of MCAS patients. Since only a small number of SM patients were included, only rough estimates of these sensitivities are possible for this group of MCAD patients, which may account at least partly for why we found sTryp > 20 ng/ml in only 53% of the SM patients in this study compared to about 80–85% found in previous studies ([16]; further references therein).

### Plasma heparin levels in MCAD patients

In human tissues, MCs (of both the connective tissue and mucosal varieties; [17, 18]) are the main source of heparin [19, 20]. Human MCs have been estimated to contain 2.4 to 7.8 μg heparin per 10^6^ cells [21]. The only other known endogenous source of heparin in humans is the basophil, which appears to produce far less heparin. Hence, in the absence of heparin therapy or rare basophil disease, elevated pHL fairly specifically reflects MC activation In the present study pHL was elevated in 41% of MCAS patients; in investigations of healthy individuals, no pHL elevation was detected. (present study; [22]) Basal pHL seems to reflect the basal activation state of the MCs in the organism. As a means of non-pharmacologic activation of MCs, venous obstruction for 10 minutes increased the sensitivity of elevated pHL as an indicator for pathological MC activation in MCAS patients to 59% and in SM patients to 47%, while post-obstruction pHL levels in normal individuals remained normal. These findings suggest that this simple, brief venous occlusion test is a useful indicator of the presence of pathologically irritable MCs, at least in the obstructed compartment of the body.

### Serum tryptase measurements

Serum tryptase has been shown repeatedly to be elevated to a level of > 20 ng/ml in the majority of SM patients (for references, see Table 1). sTryp was elevated above reference in 73% of the SM patients in this study (and elevated > 20 ng/ml in 53% of the SM patients) but was clearly less suited to indicate MC activation in MCAS patients (sensitivity only 10%), similar to previous findings (Table 1). These findings are consistent with contemporary understanding that sTryp far dominantly reflects total body MC load and far less MC activation state [23, 24]. The specificity, too, of sTryp can be adversely affected by elevated levels found in hematologic non-MC-lineage neoplasms and end-stage kidney disease. [23, 25, 26]

### Histamine and histamine metabolites in plasma and urine

An established approach to estimate systemic MC activation is the measurement of histamine and/or its metabolites in plasma and urine [27–30]. However, histamine is also expressed in (and released from) basophils, and histamine levels depend on many variables such as food and drug intake. Nevertheless, in a patient with apparent mediator-related symptoms, elevated levels of histamine or its metabolites may serve to confirm MC and/or basophil activation (Table 1). In the present study the sensitivity of uNMH for indicating increased MC activation was low in MCAS patients (22%) and intermediate in SM patients (43%), similar to previous findings (Table 1).

### Serum chromogranin A determination

Chromogranin A is another MC product [31–33] that can be used as a fairly specific marker for MC activation in the absence of cardiac and renal failure, neuroendocrine cancer, and proton pump inhibitor use. Recently, in an American cohort of 122 MCAD patients, elevated sCgA serum levels were found in 17%. [11]. The findings in the present investigation are similar, with a sensitivity of 23% for all MCAD patients (much better in SM than MCAS, 63% vs. 12%).

### Potential utility of certain prostaglandin derivatives in detecting MCAD

Although also produced in macrophages, Langerhans cells, liver endothelium, platelets, Th2 helper T cells, stimulated osteoblasts, and possibly adipocytes, PGD_2_ appears to be far dominantly produced in MCs (for review, see [11]), yielding attractive specificity for clinical detection of MCAD. However, PGD_2_ is metabolized so rapidly that its measured levels may substantially underestimate its total production. The PGD_2_ metabolite 9α,11β-PGF_2α_ is more stable than the parent compound [34], leading some to preferentially test such metabolites over PGD_2_. Recently, Ravi et al. reported that the determination of a 24-hour urine 9α,11β-PGF_2α_ level is helpful to identify patients with MCAS [35]. Elevated levels were detected in 68% of 25 patients investigated [35]. Hence, its sensitivity is close to that of post-occlusion pHL (59%). Excretion of 9α,11β-PGF_2α_ was clearly reduced after intake of non-steroidal anti-inflammatory drugs (NSAIDs), which inhibit cyclooxygenase and thus limit PG production, PGD_2_ included. Therefore, the patient’s history of use of NSAIDs must be taken into account when planning the laboratory assessment for PG levels. In addition to a direct determination of PGD_2_ and/or its metabolite 9α,11β-PGF_2α_ we have recently introduced an *in vitro* test in which substance-P-triggered release of PGD_2_ from peripheral blood leukocytes unambiguously distinguishes MCAD patients from healthy individuals [36].

### Absent correlation of mediator levels

We found no correlation among any sets of the mediators we tested, suggesting that MCs often release their mediators selectively, i.e., via multiple trigger-dependent processes of differential mediator release [37 38].

## Conclusions

Our data reveal that elevated pHL is a useful indicator of increased systemic MC activation. In particular, in patients with MCAS its sensitivity is superior to the sensitivities of the other mediators investigated here. Thus, pHL is a reasonable addition to the repertoire of MC mediator testing which can help objectively substantiate a diagnosis of MCAD. Elevated post-venous-occlusion pHL likely suggests presence of pathologically activated MCs in the obstructed compartment. The finding that combined sensitivity of the mediators was clearly higher (at least in the SM group) than the sensitivities of each mediator alone (Table 5) suggests an advantage to determining more rather than fewer MC mediator levels in a patient suspected to have MCAD [11]. The diagnostician must remain aware, though, of the many factors affecting MC mediator levels *in vivo* and *in vitro*. Increased systemic MC activation is considered present if an elevated MC mediator level can be found. However, given the above-noted challenges in detecting elevated levels of MC mediators, some patients present normal initial laboratory results in spite of medical histories typical for MCAD. When repeated efforts to identify aberrant MC activation using the above-described mediators fail, consideration can be given to screening for aberrant expression of other mediators such as prostaglandin D_2_ or its more stable metabolite 9α,11β-PGF_2α_ or somewhat less specific mediators such as certain leukotrienes, Factor VIII, plasma free norepinephrine, tumor necrosis factor alpha, and interleukin-6 (for review, see [11]).

## Supporting Information

S1 Table Determined mast cell mediator levels. (XLS)

## References

[1] Molderings GJ, Brettner S, Homann J, Afrin LB. Mast cell activation disease: a concise practical guide for diagnostic workup and therapeutic options. J Hematol Oncol. 2011;4: 10 10.1186/1756-8722-4-10 21418662 PMC3069946

[2] Molderings G. The genetic basis of mast cell activation disease—looking through a glass darkly. Crit Rev Oncol/Hematol. 2015;93: 75–89. 10.1016/j.critrevonc.2014.09.001 25305106

[3] Hamilton MJ, Hornick JL, Akin C, Castells MC, Greenberger NJ. Mast cell activation syndrome: A newly recognized disorder with systemic clinical manifestations. J Allergy Clin Immunol. 2011;128: 147–152. 10.1016/j.jaci.2011.04.037 21621255

[4] Valent P, Akin C, Arock M, Brockow K, Butterfield JH, Carter MC, et al Definitions, criteria and global classification of mast cell disorders with special reference to mast cell activation syndromes: A consensus proposal. Int Arch Allergy Immunol. 2012;157: 215–225. 10.1159/000328760 22041891 PMC3224511

[5] Valent P, Horny HP, Escribano L, Longley BJ, Li CY, Schwartz LB, et al Diagnostic criteria and classification of mastocytosis: a consensus proposal. Leuk Res. 2001;25: 603–625. 11377686 10.1016/s0145-2126(01)00038-8

[6] Valent P, Akin C, Escribano L, Födinger M, Hartmann K, Brockow K, et al Standards and standardization in mastocytosis: consensus statements on diagnostics, treatment recommendations and response criteria. Eur J Clin Invest. 2007;37: 435–453. 17537151 10.1111/j.1365-2362.2007.01807.x

[7] Haenisch B, Nöthen MM, Molderings GJ. Systemic mast cell activation disease: the role of molecular genetic alterations in pathogenesis, heritability and diagnostics. Immunology. 2012;137: 197–205. 10.1111/j.1365-2567.2012.03627.x 22957768 PMC3482677

[8] Cohen SS, Skovbo S, Vestergaard H, Kristensen T, Møller M, Bindslev-Jensen C, et al Epidemiology of systemic mastocytosis in Denmark. Br J Haematol. 2014;166: 521–528. 10.1111/bjh.12916 24761987

[9] van Doormaal JJ, Arends S, Brunekreeft KL, van der Wal VB, Sietsma J, van Voorst Vader PC, et al Prevalence of indolent systemic mastocytosis in a Dutch region. J Allergy Clin Immunol. 2013;131: 1429–1431. 10.1016/j.jaci.2012.10.015 23219169

[10] Molderings GJ, Haenisch B, Bogdanow M, Fimmers R, Nöthen MM. Familial occurrence of systemic mast cell activation disease PLoS One. 2013;8: e76241 10.1371/journal.pone.0076241 24098785 PMC3787002

[11] Afrin LB, Molderings GJ. A concise, practical guide to diagnostic assessment for mast cell activation disease. World J Hematol. 2014;3: 1–17. 10.1186/1756-8722-4-10 PMC3069946 21418662

[12] Seidel H, Molderings GJ, Oldenburg J, Meis K, Kolck UW, Homann J, et al Bleeding diathesis in patients with mast cell activation disease. Thromb Haemost. 2011;106: 987–989. 10.1160/TH11-05-0351 21901238

[13] Alfter K, von Kügelgen I, Haenisch B, Frieling T, Hülsdonk A, Haars U, et al New aspects of liver abnormalities as part of the systemic mast cell activation syndrome. Liver Int. 2009;29: 181–186. 10.1111/j.1478-3231.2008.01839.x 18662284

[14] Yang W, Chen J, Zhou L. Effects of shear stress on intracellular calcium change and histamine release in rat basophilic leukemia (RBL-2H3) cells. J Environ Pathol Toxicol Oncol. 2009;28: 223–230. 19888909 10.1615/jenvironpatholtoxicoloncol.v28.i3.30

[15] Fowlkes V, Wilson CG, Carver W, Goldsmith EC. Mechanical loading promotes mast cell degranulation via RGD-integrin dependent pathways. J Biomech. 2013;46: 788–795. 10.1016/j.jbiomech.2012.11.014 23261248 PMC3646572

[16] van Doormaal JJ, van der Veer E, van Voorst Vader PC, Kluin PM, Mulder AB, van der Heide S, et al Tryptase and histamine metabolites as diagnostic indicators of indolent systemic mastocytosis without skin lesions. Allergy. 2012;67: 683–690. 10.1111/j.1398-9995.2012.02809.x 22435702

[17] Craig SS, Irani AM, Metcalfe DD, Schwartz LB. Ultrastructural localization of heparin to human mast cells of the MCTC and MCT types by labeling with antithrombin III-gold. Lab Invest. 1993;69: 552–561 8246447

[18] Rönnberg E, Melo FR, Pejler G. Mast cell proteoglycans. J Histochem Cytochem. 2012;60: 950–62. 10.1369/0022155412458927 22899859 PMC3527880

[19] Studer A. Occurrence and significance of endogenous heparin. Experientia. 1954;10: 148–152. 13161899 10.1007/BF02158526

[20] Jaques LB. Endogenous heparin. Semin Thromb Hemost. 1978;4: 326–349. 97786 10.1055/s-0028-1087139

[21] Metcalfe DD, Lewis RA, Silbert JE, Rosenberg RD, Wasserman SI, Austen KF. Isolation and characterization of heparin from human lung. J Clin Invest. 1979;64: 1537–1543. 500822 10.1172/JCI109613 PMC371305

[22] Władysław S. Endogenous heparin—a protective marker in patients with myocardial infarction, Coron. Artery Dis. 2002;13: 423–426. 12544717 10.1097/00019501-200212000-00007

[23] Sperr WR, Jordan JH, Fiegl M, Escribano L, Bellas C, Dirnhofer S, et al Serum tryptase levels in patients with mastocytosis: correlation with mast cell burden and implication for defining the category of disease. Int Arch Allergy Immunol. 2002;128: 136–141. 12065914 10.1159/000059404

[24] Quintás-Cardama A, Sever M, Cortes J, Kantarjian H, Verstovsek S. Bone Marrow Mast cell burden and serum tryptase level as markers of response in patients with systemic mastocytosis. Leuk Lymphoma. 2013;54: 1959–1964. 10.3109/10428194.2012.763121 23278641 PMC5127201

[25] Schwartz LB. Diagnostic value of tryptase in anaphylaxis and mastocytosis. Immunol Allergy Clin North Am. 2006;26: 451–463. 16931288 10.1016/j.iac.2006.05.010

[26] Sirvent AE, González C, Enríquez R, Fernández J, Millán I, Barber X, et al Serum tryptase levels and markers of renal dysfunction in a population with chronic kidney disease. J Nephrol. 2010;23: 282–290. 20349428

[27] Hogan AD, Schwartz LB. Markers of mast cell degranulation. Methods. 1997;13: 43–52. 9281467 10.1006/meth.1997.0494

[28] Akin C, Metcalfe DD. Surrogate markers of disease in mastocytosis. Int Arch Allergy Immunol. 2002;127: 133–136. 11919423 10.1159/000048184

[29] Di Lorenzo G, Pacor ML, Vignola AM, Profita M, Esposito-Pellitteri M. Urinary metabolites of histamine and leukotrienes before and after placebo-controlled challenge with ASA and food additives in chronic urticaria patients. Allergy. 2002;57: 1180–1186. 12464047 10.1034/j.1398-9995.2002.23767.x

[30] van Toorenenbergen AW, Oranje AP. Comparison of serum tryptase and urine N-methylhistamine in patients with suspected mastocytosis. Clin Chim Acta. 2005;359: 72–77. 15913591 10.1016/j.cccn.2005.03.041

[31] Ibelgaufts H. Mast Cells. In: COPE: Cytokines and Cells Online Pathfinder Encyclopaedia. Available: http://www.copewithcytokines.de/cope.cgi?key=mast%20cells. Accessed September 3, 2013.

[32] Prasad P, Yanagihara AA, Small-Howard AL, Turner H, Stokes AJ. Secretogranin III directs secretory vesicle biogenesis in mast cells in a manner dependent upon interaction with chromogranin A. J Immunol. 2008;181: 5024–5034. 18802106 10.4049/jimmunol.181.7.5024

[33] Stefanov S, Vodenicharov AP, Gulubova MV. Immunocytochemical expression of Chromogranin A in mast cells in the canine paranasal sinus. Revue Méd Vét. 2013;164: 453–456. 10.1016/j.bulcan.2015.01.010 25758301

[34] Bochenek G, Niżankowska E, Gielicz A, Świerczyńska M, Szczeklik A. Plasma 9α,11β-PGF_2_, a PGD_2_ metabolite, as a sensitive marker of mast cell activation by allergen in bronchial asthma. Thorax. 2004;59: 459–464. 15170023 10.1136/thx.2003.013573 PMC1747024

[35] Ravi A, Butterfield J, Weiler CR. Mast cell activation syndrome: improved identification by combined determinations of serum tryptase and 24-hour urine 11ß-prostaglandin2α. J Allergy Clin Immunol Pract. 2014;2: 775–778. 10.1016/j.jaip.2014.06.011 25439370

[36] Schäfer D, Dreßen P, Brettner S, Rath NF, Molderings GJ, Jensen K, et al Prostaglandin D_2_-supplemented "functional eicosanoid testing and typing" assay with peripheral blood leukocytes as a new tool in the diagnosis of systemic mast cell activation disease: an explorative diagnostic study. J Transl Med. 2014;12: 213 10.1186/s12967-014-0213-2 25113638 PMC4283146

[37] Theoharides TC, Bondy PK, Tsakalos ND, Askenase PW. Differential release of serotonin and histamine from mast cells. Nature. 1982;297: 229–231. 6176873 10.1038/297229a0

[38] Theoharides TC, Kempuraj D, Tagen M, Conti P, Kalogeromitros D. Differential release of mast cell mediators and the pathogenesis of inflammation. Immunol Rev. 2007;217: 65–78. 17498052 10.1111/j.1600-065X.2007.00519.x

[39] Pardanani A, Chen D, Abdelrahman RA, Reichard KK, Zblewski D, Wood AJ, et al Clonal mast cell disease not meeting WHO criteria for diagnosis of mastocytosis: clinicopathologic features and comparison with indolent mastocytosis. Leukemia. 2013;27: 2091–2094. 10.1038/leu.2013.227 23896642

[40] Kassaba D, Koterbaa A, Jianga Y, Akin A. Elevated baseline tryptase levels in patients with mast cell activation syndromes without evidence of mastocytosis. J Allergy Clin Immunol. 2008;121(Suppl 1): S67.

[41] Alvarez-Twose I, González de Olano D, Sánchez-Muñoz L, Matito A, Esteban-López MI, Vega A, et al Clinical, biological, and molecular characteristics of clonal mast cell disorders presenting with systemic mast cell activation symptoms. J Allergy Clin Immunol. 2010;125: 1269–1278. 10.1016/j.jaci.2010.02.019 20434205

[42] Butterfield JH, Weiler CR. Prevention of mast cell activation disorder-associated clinical sequelae of excessive prostaglandin D(2) production. Int Arch Allergy Immunol. 2008;147: 338–343. 10.1159/000144042 18622141

[43] Frieling T, Meis K, Kolck UW, Homann J, Hülsdonk A, Haars U, et al Evidence for mast cell activation in patients with therapy-resistant irritable bowel syndrome. Z Gastroenterol. 2011;49: 191–194. 10.1055/s-0029-1245707 21298604

[44] Ravi A, Butterfield JH, Weiler CR. Mast cell activation syndrome associated with elevation in serum tryptase or 24-hour urine 11ß-prostaglandin-F2α. J Allergy Clin Immunol. 2013;131: AB117. 10.1016/j.jaip.2014.06.011 25439370

[45] Sargur R, Cowley D, Murng S, Wild G, Green K. Raised tryptase without anaphylaxis or mastocytosis: heterophilic antibody interference in the serum tryptase assay. Clin Exp Immunol. 2011;162: 339–345. 10.1111/j.1365-2249.2010.04287.x PMC3048617 21303361

[46] Afrin LB. Polycythemia from mast cell activation syndrome: lessons learned. Am J Med Sci. 2011;342: 44–49. 10.1097/MAJ.0b013e31821d41dd 21642812

[47] Akhavein MA, Patel NR, Muniyappa PK, Glover SC. Allergic mastocytic gastroenteritis and colitis: an unexplained etiology in chronic abdominal pain and gastrointestinal dysmotility. Gastroenterol Res Pract. 2012;2012: 950582 10.1155/2012/950582 22577375 PMC3346686

[48] Kushnir-Sukhov NM, Brittain E, Scott L, Metcalfe DD. Clinical correlates of blood serotonin levels in patients with mastocytosis. Eur J Clin Invest. 2008;38: 953–958. 10.1111/j.1365-2362.2008.02047.x 19021721 PMC3795418

[49] Butterfield J, Weiler C. Whole blood serotonin levels in cutaneous mastocytosis, systemic mastocytosis and mast cell activation syndroms. J Allergy Clin Immunol. 2011: AB132.

[50] Molderings GJ. Physiological, pathophysiological and therapeutic impact of the enteric serotonergic system. Arzneimittelforschung. 2012;62: 157–166. 10.1055/s-0032-1306321 22438071

[51] Butterfield JH. Increased leukotriene E4 excretion in systemic mastocytosis. Prostaglandins Other Lipid Mediat. 2010;92: 73–76. 10.1016/j.prostaglandins.2010.03.003 20380889

[52] Raithel M, Zopf Y, Kimpel S, Naegel A, Molderings GJ, Buchwald F, et al The measurement of leukotrienes in urine as diagnostic option in systemic mastocytosis. J Physiol Pharmacol. 2011;62: 469–472. 22100848

[53] Dahlén SE, Kumlin M. Monitoring mast cell activation by prostaglandin D2 in vivo. Thorax. 2004;59: 453–455. 15170020 10.1136/thx.2004.026641 PMC1747045

[54] O’Sullivan S. On the role of PGD2 metabolites as markers of mast cell activation in asthma. Acta Physiol Scand. 1999;644(Suppl): 1–74. 10352758

[55] Luna-Gomes T, Magalhães KG, Mesquita-Santos FP, Bakker-Abreu I, Samico RF, Molinaro R, et al Eosinophils as a novel cell source of prostaglandin D2: autocrine role in allergic inflammation. J Immunol.2011;187: 6518–6526. 10.4049/jimmunol.1101806 22102725 PMC3237921

[56] Butterfield JH. Survey of aspirin administration in systemic mastocytosis. Prostaglandins Other Lipid Mediat. 2009;88: 122–124. 10.1016/j.prostaglandins.2009.01.001 19429499

[57] Morrow JD, Guzzo C, Lazarus G, Oates JA, Roberts LJ 2nd. Improved diagnosis of mastocytosis by measurement of the major urinary metabolite of prostaglandin D2. J Invest Dermatol. 1995;104: 937–940. 7769262 10.1111/1523-1747.ep12606209

